# Our Greatest (and Most Neglected) National Asset

**Published:** 1908-03

**Authors:** 


### EDITORIAL DEPARTMENT.

### OUR GREATEST (AND MOST NEGLECTED) NATIONAL ASSET.

[I reproduce from the *Houston Post* of February 15th, ult., a letter addressed by the editor of this JOURNAL to the press and people of Texas. Read it. It is self-explanatory. A copy was furnished simultaneously to each of the great dailies of the State. Some of them published it; some of them did not. One leading daily published it with the paragraph on patent medicines stricken out. The managing editor of that paper told me frankly that he would not antagonize the patent medicine interest, as his paper couldn't live a month without their patronage. There is not a 14-year-old girl in Texas who does not know what "Tansy wafers" stands for. Think of it!

Such a situation is deplorable; and the magnitude of the evils and the necessity for government action, swift and sure, are only emphasized. When leading papers of a great State like Texas are so dominated by "interests" destructive and far-reaching as those of the saloon and patent medicines are shown to be, that the owners *dare not* enlighten the people; when one, representing and speaking for a vast body of the most learned men in America can not be heard in such a cause, it is a sad commentary on the degeneracy of the times, and a disgrace to our civilization.]

AUSTIN, TEXAS, February 15, 1908.

(*Staff Correspondent of the Post.*)

"The following has been given out here by F. E. Daniel, M. D., editor TEXAS MEDICAL JOURNAL, late President American International Congress on Tuberculosis, New York, 1906, and late President of the State Medical Association of Texas:

*To the Press and People of Texas:*

The Association for the Advancement of Science is composed of the ablest and most distinguished men in America, in every branch of learning. It includes sanitarians and other scientists, educators, authors, engineers, editors, ministers and bishops, political economists, the heads of the great universities, philanthropists, philosophers, statesmen and publicists. This association has a committee of one hundred on National health, and they have a subcommittee on magazine publicity; also an allied organization called the Author's League. As a member of this league, on their behalf and by request of the executive committee, I write this letter to the press.

Professor J. Pease Norton, of Yale, Executive Secretary, writes me: "The committee of one hundred is seeking to awaken public interest in the question of Federal regulation of public health to the end that Congress may enact effective legislation looking to such regulation." In a memorable address before the association last year he said:

"The salvation of the civilization and of the race lies in the hands of exceptional men. The hope of the race rests in the efficient organization for action. * * * There are four great wastes of our civilization, the more lamentable because they are unnecessary. They are preventable deaths, preventable sickness, preventable conditions of low mental and physical efficiency, and preventable ignorance." He then instances consumption as one of the factors in this great and needless waste, and says: "We look with horror on the black plague of the Middle Ages. The black plague was but a passing cloud compared with the white waste visitation. Of the people living today over 8,000,000 will die of tuberculosis, and the Federal government does not raise a hand to help them."

The object of this letter is to enlist the press in this great reform movement for humanity and race integrity. "Our National health is physically our greatest National asset," says President Roosevelt in a letter to the committee of one hundred of the Association for the Advancement of Science. It is our greatest asset not only physically, but in every other respect—economically, socially, commercially, morally, mentally and collectively—both national, State and municipal. It is the fundamental and most important interest of a people, a State or a nation, the interest upon which all other interests depend. That nation is not the strongest that has the largest population, for with races as with individuals the strongest and fittest survive, but the one whose units are physically and mentally sound. Yet, strange to say, this greatest and most far-reaching and vital interest is less safeguarded by government than any other, public or private, personal or property.

### IGNORANCE OF CONDITIONS.

This paradox is not the result of indifference, but it is because the people and our lawmakers do not know the conditions which are in operation to the detriment of the individual and the deterioration of the race, many of the factors of which are preventable and can be removed. Nor do they know the value and scope of sanitary science in preventing and removing those factors. They have not thought and reflected upon the subject, notwithstanding the recent and brilliant example of the prevention of yellow fever by exterminating the fever-producing mosquito, and the conversion of the Panama isthmus country from the most deadly region on earth to one of salubrity, where life is not only possible, but pleasant. And it is almost impossible to educate the people or to get the lawmakers to take any interest in sanitary legislation, however vital. There are so many other things to do that evils which are productive of fearful waste of life and which in the cause of humanity cry aloud for reform are overlooked and neglected, or because the very magnitude of the needed reform discourages all attempts to deal with it. True, efforts are made both by Congress and State Legislatures to deal with certain phases of the subject, but it is inadequate and spasmodic. What is wanted and necessary is a comprehensive law of Congress, supplemented by efficient State laws, covering the entire field of sanitation, and the removal by the strong arm of authority and the strong purse of the general government of the evils which are not only destroying our people by the hundreds of thousands annually, but which are affecting our race integrity in the diminution of the birth rate and the production of feebler and still feebler offspring generation after generation.

I instance consumption, a preventable disease. It destroys 150,000 American people every year (411 every day, one every three minutes; United States census reports). And yet its spread from sick to well is preventable.

I instance the increase of insanity in Texas from 1860 to 1904 from 50 patients to 5000, the cost of maintenance from $12,000 a year in 1860 to $784,000 a year in 1904 (Comptroller's figures), the rate of increase being 6800 per cent as against 504 per cent of population. Or, in other words, the insane have increased 13.7 times faster than the population (Professor M. L. Graves, University of Texas, late Superintendent Southwestern Insane Asylum); and this increase, according to the published reports of our asylum superintendents, is due directly and indirectly to alcohol in from 50 to 95 per cent of cases. The government, State and national, licenses the sale of an agent thus fearfully destructive of life and health, producing also crime, pauperism and prostitution.

This letter is a plea for removing by the government, in so far as it is possible to do so, of every agency that can be shown (as in the far-reaching influences of licensed saloons and alcoholic patent medicines) to be swift factors in race degeneracy. Yet no Legislature will license saloons for the indiscriminate sale of morphine, opium or cocaine, not more destructive.

I instance also the extensive use of drinking water polluted by animal poisons; human excrement, reeking with the germs of typhoid fever, diphtheria, dysentery and other preventable diseases which carry off their scores of hundreds of our citizens.

### PATENT MEDICINES.

I instance another one of the greatest evils, one which is far-reaching in its destructive influences, one which flourishes upon the ignorance and credulity of the people. It is the use and abuse of the accursed patents medicines. Many of these contain alcohol, opium, chloral or cocaine. Our daily press, and especially the religious press, fosters and promotes its "interests" by advertisements, many of which are fraudulent on their face and most of which hold out false and delusive hopes by specious promises; many are thinly veiled abortifacients, the use of which should be prohibited by law. The Federal government should deal with this monster evil.

It seems to the writer that the greatest and most imperative duty that the government, State and National, owes to its people is to protect them from these and all other dangers, and especially from the ravages of preventable diseases. To that end National legislation is necessary. It is thought the magnitude and great importance of the problem justifies and demands a National department of public health with a secretary or commissioner in the President's cabinet. In this department there should be separate bureaus covering and dealing with the subdivisions in sanitation; for instance, a bureau of education and schools, of infant hygiene (the greatest mortality is among infants); of general sanitation (under which would come the prevention of water pollution, milk infection, the breeding of flies, local epidemics, etc.); of pure foods and drugs and beverages; of registration (physicians, midwives, drugs and druggists); of quarantine, of immigration, of health information, and, fundamentally, a bureau of vital statistics, etc.

### LEGISLATION FOR SANITATION.

Legislation in the interest of public health is legislation in the interest of public morals and of social economics; for what affects the health of a people adversely affects also the morals and political economies, producing crime, insanity, pauperism and prostitution, and fewer and feebler progeny. It may be that sooner than we expect America will be called upon to measure strength with a virile people who understand and practice sanitary science and who have recently applied its principles in war with such success as to practically nullify the great numerical superiority of their antagonist. That the general government should at once take vigorous control of the situation, and by the enactment and rigid enforcement of a sanitary code, backed by an adequate appropriation, is demanded by every consideration of propriety, public safety, self-defense and National existence. Inordinate wealth, luxury, debauchery, idleness and neglect of the right principles of living render a race unfit, and in the history of the world these have preceded the downfall of the greatest civilizations.

On behalf of my distinguished colleagues and in the name of humanity and patriotism, I call on the press of Texas to lend their great power toward awakening an appreciative interest in this great reform. I trust that our great dailies will take it up editorially and urge prompt action by Congress. Professor J. P. Norton, of Yale, Executive Secretary, drawer 30, New Haven, Conn., will gladly furnish statistics and other literature free of charge, on request.

